# The formyl peptide fMLF primes platelet activation and augments thrombus formation

**DOI:** 10.1111/jth.14466

**Published:** 2019-05-24

**Authors:** Maryam F. Salamah, Divyashree Ravishankar, Rajendran Vaiyapuri, Leonardo A. Moraes, Ketan Patel, Mauro Perretti, Jonathan M. Gibbins, Sakthivel Vaiyapuri

**Affiliations:** ^1^ School of Pharmacy University of Reading Reading UK; ^2^ School of Pharmacy University of Reading Malaysia Johor Malaysia; ^3^ School of Biological Sciences University of Reading Reading UK; ^4^ William Harvey Research Institute, Queen Mary University of London London UK

**Keywords:** fMLF, formyl peptide receptors, hemostasis, platelets, thrombosis

## Abstract

**Essentials**

- The role of formyl peptide receptor 1 (FPR1) and its ligand, fMLF, in the regulation of platelet function, hemostasis, and thrombosis is largely unknown.
- *Fpr1*‐deficient mice and selective inhibitors for FPR1 were used to investigate the function of fMLF and FPR1 in platelets.
- N‐formyl‐methionyl‐leucyl‐phenylalanine primes platelet activation and augments thrombus formation, mainly through FPR1 in platelets.
- Formyl peptide receptor 1 plays a pivotal role in the regulation of platelet function.

**Background:**

Formyl peptide receptors (FPRs) play pivotal roles in the regulation of innate immunity and host defense. The FPRs include three family members: FPR1, FPR2/ALX, and FPR3. The activation of FPR1 by its high‐affinity ligand, *N*‐formyl‐methionyl‐leucyl‐phenylalanine (fMLF) (a bacterial chemoattractant peptide), triggers intracellular signaling in immune cells such as neutrophils and exacerbates inflammatory responses to accelerate the clearance of microbial infection. Notably, fMLF has been demonstrated to induce intracellular calcium mobilization and chemotaxis in platelets that are known to play significant roles in the regulation of innate immunity and inflammatory responses. Despite a plethora of research focused on the roles of FPR1 and its ligands such as fMLF on the modulation of immune responses, their impact on the regulation of hemostasis and thrombosis remains unexplored.

**Objective:**

To determine the effects of fMLF on the modulation of platelet reactivity, hemostasis, and thrombus formation.

**Methods:**

Selective inhibitors for FPR1 and *Fpr1*‐deficient mice were used to determine the effects of fMLF and FPR1 on platelets using various platelet functional assays.

**Results:**

*N*‐formyl‐methionyl‐leucyl‐phenylalanine primes platelet activation through inducing distinctive functions and enhances thrombus formation under arterial flow conditions. Moreover, FPR1 regulates normal platelet function as its deficiency in mouse or blockade in human platelets using a pharmacological inhibitor resulted in diminished agonist‐induced platelet activation.

**Conclusion:**

Since FPR1 plays critical roles in numerous disease conditions, its influence on the modulation of platelet activation and thrombus formation may provide insights into the mechanisms that control platelet‐mediated complications under diverse pathological settings.

## 1 INTRODUCTION

Platelets are small circulating blood cells that play indispensable roles in the regulation of hemostasis to prevent excessive bleeding upon vascular injury. However, their unwarranted activation under pathological conditions leads to the formation of blood clots (thrombi) within the circulation.^1^ This results in reduced/retarded blood supply to vital organs including the heart and brain, which leads to heart attacks or strokes, respectively.^2^ Moreover, platelet activation during microbial infection results in their aggregation, thrombus formation in the microvasculature, and, in later stages, sequestration of platelets in organs such as the lungs, instigating thrombocytopenia and bleeding complications.^3^ In addition to their prominent roles in hemostasis and thrombosis, platelets play a crucial role in the regulation of innate immunity, inflammatory responses, and clearance of microbial infection.^4^, ^5^, ^6^ Platelets contain a broad spectrum of receptors that induce inflammatory responses during microbial infection and other pathological conditions. In addition, platelets secrete various inflammatory and immunomodulatory molecules from their granules upon activation. They also possess antimicrobial proteins including thrombocidins, cathelicidins, and human β‐defensins that trigger direct microbicidal activities.^4^ Furthermore, platelets can directly bind and internalize invading microbes.^7^ The presence of major inflammatory molecules such as formyl peptide receptors (FPRs) and toll‐like receptors facilitates platelets to recognize a diverse array of endogenous damage‐associated molecular patterns and exogenous pathogen‐associated molecular patterns. Collectively, these properties render platelets effector and sentinel cells in primary host defense against invading pathogenic microbes.^7^, ^8^

The FPRs belong to a family of G protein‐coupled receptors and are predominantly expressed in immune cells, where they play a prominent role in the regulation of inflammatory responses and host defense. In humans, three FPR family members have been identified: FPR1, FPR2/ALX, and FPR3.^9^ Although they were originally identified by their capability to recognize *N*‐formyl peptides produced from bacteria or mitochondria of damaged cells, FPRs can bind a wide variety of structurally and functionally diverse ligands. These include bacterial and mitochondrial formyl peptides, nonformylated peptides/proteins, and small lipid molecules.^10^ While FPR1 binds to bacterial‐derived *N*‐formyl peptides with high affinity, FPR2/ALX largely binds to mitochondrial formyl peptides.^9^, ^11^ The ligation of *N*‐formyl peptides to FPRs in immune cells triggers a range of signaling cascades resulting in numerous biological activities. For example, the stimulation of FPR1 by fMLF in neutrophils induces degranulation, chemotaxis, production of superoxide anions, calcium mobilization, cytokine release, and expression of various surface markers.^12^, ^13^ During microbial infection, invading bacteria release *N*‐formyl peptides that facilitate the recruitment of immune cells to the site of infection and accelerate the clearance of microbial infection and repair of tissue damage.^14^

Czapiga et al^15^ reported the presence of FPR1 in platelets and its ability to induce chemotactic and migratory responses upon ligation with *N*‐formyl peptides, which emphasize a crucial role for FPR1 in platelet‐mediated immune responses. Notably, bacterial or synthetic fMLF has been shown to act as a potent chemotactic agent through FPR1 in platelets. Despite numerous reports on the immune functions of FPR1, its impact upon ligation with fMLF on the modulation of hemostasis and thrombosis remains uncharacterized. Recently, we have reported the presence of FPR2/ALX in human platelets and its significance in the regulation of LL37‐induced platelet activation and thrombus formation.^16^ Here, we report the ability of fMLF to prime platelets and augment thrombus formation, and the significance of FPR1 in the regulation of platelet function in the presence and absence of fMLF.

## 2 METHODS

### 2.1 Preparation of human platelet‐rich plasma and isolated platelets

The University of Reading Research Ethics Committee approved all the experimental procedures using human blood from healthy volunteers. The blood samples were collected from healthy, aspirin‐free volunteers after obtaining written informed consent. The blood was collected into VACUETTE blood collection tubes containing 3.2% (w/v) sodium citrate. The blood samples were then centrifuged at 102 *g* for 20 minutes at room temperature to obtain platelet‐rich plasma (PRP). The PRP was rested at 30°C for 30 minutes prior to use. For the preparation of isolated platelets, the blood was mixed with ACD [2.5% (w/v) sodium citrate, 2% (w/v) D‐glucose, and 1.5% (w/v) citric acid] at 1 (ACD): 9 (blood) ratio and centrifuged at 102 *g* for 20 minutes. The PRP was collected, mixed with appropriate volume of ACD and prostaglandin I_2_ (PGI_2_), and centrifuged at 1413 *g* for 10 minutes at room temperature. The resultant platelet pellet was resuspended in modified Tyrodes‐HEPES buffer (134 mmol/L NaCl, 2.9 mmol/L KCl, 0.34 mmol/L Na_2_HPO_4_.12H_2_O, 12 mmol/L NaHCO_3_, 20 mmol/L HEPES, and 1 mmol/L MgCl_2_, pH 7.3) with appropriate volume of ACD and PGI_2_ and centrifuged once again at 1413 *g* for 10 minutes at room temperature. The resultant platelet pellet was resuspended to a final density of 4 × 10^8^ cells/mL in modified Tyrode's‐HEPES buffer and allowed to rest for 30 minutes at 30°C prior to use.

### 2.2 Mouse blood collection and platelet preparation

The mouse strains of *Fpr1* ^−/−^ 17 and *Fpr2/3* ^−/−^ 18 on a C57BL/6 background obtained from William Harvey Research Institute, London, UK, and wild‐type C57BL/6 mice from Envigo, UK, were used in this study. The mice were sacrificed with CO_2_ and the blood was directly collected by cardiac puncture into a syringe containing 3.2% (w/v) sodium citrate at 1 (citrate):9 (blood) ratio. The blood was then centrifuged at 203 *g* for 8 minutes at room temperature and the PRP was collected. The remaining blood was resuspended in 500 μL of modified Tyrode's‐HEPES buffer and centrifuged once again at 203 *g* for 5 minutes. The resultant PRP with PGI_2_ then centrifuged at 1028 *g* for 5 minutes. The resultant platelet pellet was resuspended in modified Tyrode's‐HEPES buffer at a density of 2 × 10^8^ cells/mL and rested for 30 minutes at 30°C prior to use.

### 2.3 In vitro thrombus formation assay

The in vitro thrombus formation assay under arterial flow conditions was performed as described previously.^19^, ^20^ Briefly, human DiOC_6_‐labeled (Sigma Aldrich) human whole blood was preincubated with a vehicle, MLF or fMLF (5 μmol/L) or cyclosporin H (CsH) (10 μmol/L), for 10 minutes before perfusion over collagen (Nycomed) (400 μg/mL)‐coated Vena8™ Biochips (Cellix Ltd) at a shear rate of 20 dynes/cm^2^. Z‐stack fluorescence images of thrombi were obtained every 30 s for up to 10 minutes using a Nikon eclipse (TE2000‐U) microscope (Nikon Instruments). The fluorescence intensity was calculated by analyzing the data using ImageJ software (National Institutes of Health).

### 2.4 Platelet adhesion assay

Platelet adhesion was measured by detecting the level of acid phosphatase in immobilized platelet lysates. One hundred microliters of collagen (10 μg/mL in 0.01 M acetic acid) were added to 96‐well plates and incubated overnight at 4°C. The unbound collagen was discarded, and the wells were blocked with 175 μL of 5% (w/v) bovine serum albumin in modified Tyrode's‐HEPES for 1 h. Plates were then washed three times with 175 μL per well of 0.1% bovine serum albumin in modifiedTyrode's‐HEPES buffer. Platelets (1 × 10^8^ cells/mL, 50 μL per well) were then added to wells and incubated at room temperature for 1 h. Nonadhered platelets were discarded, and the wells were washed three times with modified Tyrode's‐HEPES buffer. One hundred and fifty microliters of citrate lysis buffer (3.53 mmol/L p‐nitrophenyl phosphate, 71.4 mmol/L trisodium citrate, 28.55 mmol/L citric acid, 0.1% [v/v] Triton X‐100; pH 5.4) were added and the plate was incubated for 1 h at room temperature. The reaction was stopped by adding 2 M NaOH and the absorbance was measured at 405 nm using a Fluostar Optima spectrofluorimeter.

### 2.5 Tail bleeding assay

The tail bleeding assay was performed as described previously.^21^ The British Home Office has approved the experimental procedures. In brief, C57BL/6 (10‐12 weeks old; Envigo, UK) or *Fpr1* ^−/−^ mice were anesthetized using ketamine (80 mg/kg) and xylazine (5 mg/kg) administered via the intraperitoneal route and placed on a heated mat (37°C). The tail tip (3 mm) was dissected and immersed in sterile saline. The time to cessation of bleeding was measured and the assay was terminated at 20 minutes.

### 2.6 Platelet aggregation assay

The platelet aggregation assays were performed by optical aggregometry using a two‐channel platelet aggregometer (Chrono‐Log). Human isolated platelets or PRP (270 μL) were added into a siliconized cuvette and prewarmed at 37°C for 90 s. Upon addition of an agonist, the platelets were allowed to aggregate under continuous stirring at 1200 rpm for 5 minutes at 37ºC and the level of aggregation was monitored. The platelets were pretreated with different concentrations of fMLF (1, 5, 10, and 20 μmol/L) for 5 minutes before the addition of CRP‐XL (0.25 μg/mL), collagen (0.5 μg/mL), or thrombin (0.01 U/mL) and the level of aggregation was monitored. Data were analyzed by calculating the percentage of maximum platelet aggregation obtained at 5 minutes.

### 2.7 Adenosine triphosphate secretion assay

To assess the level of dense granule secretion in platelets, adenosine triphosphate (ATP) secretion was measured using a luciferin–luciferase luminescence substrate (Chrono‐Log) by lumiaggregometry (Chrono‐log). The level of ATP released from platelets upon stimulation with a platelet agonist, CRP‐XL (0.25 μg/mL), in the presence and absence of different concentrations of Boc‐MLF was measured by observing the level of luminescence released.

### 2.8 Statistical analysis

The data obtained in this study are represented as mean ± SEM. The statistical significance was analyzed using two‐tailed unpaired Student *t* test for two‐sample comparisons for the data obtained from the flow cytometric assay for FPR1 expression and platelet receptor characterization and cyclic adenosine monophosphate (cAMP) assay. For multiple comparisons such as for data obtained from in vitro thrombus formation, fMLF binding, ATP release, platelet aggregation, and activation, the statistical significance was established using 1‐way or 2‐way ANOVA followed by Bonferroni's correction. Data obtained from the tail bleeding assay were analyzed using a nonparametric Mann‐Whitney test. All statistical analyses were performed using Graphpad Prism 7 software (GraphPad Software Inc.).

## 3 RESULTS

### 3.1 Platelets express FPR1

**Figure 1:**
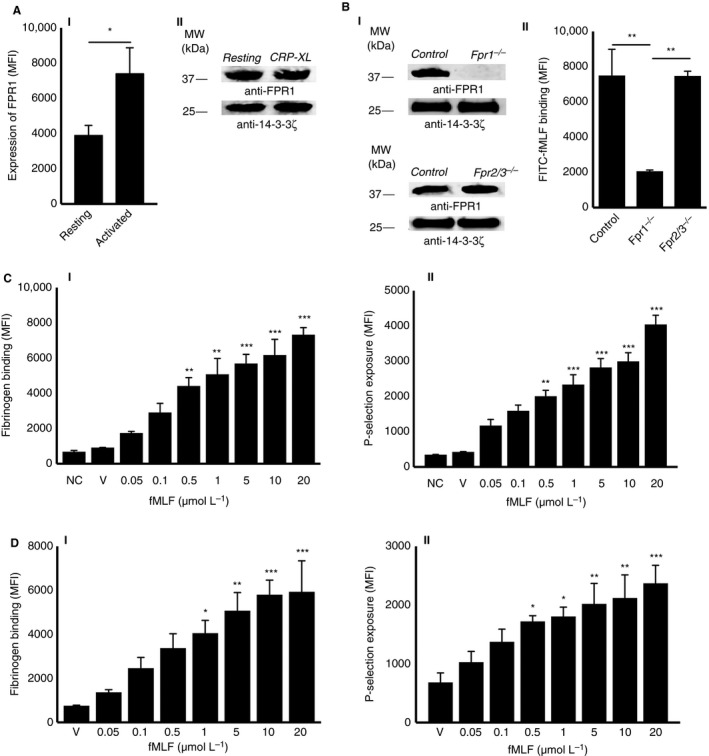
Expression of FPR1 in platelets and the impact of fMLF on platelet activation. A, the expression of FPR1 on the surface of resting or 1 μg/mL CRP‐XL‐ activated human isolated platelets was analyzed using FPR1‐selective and fluorescent‐labeled secondary antibodies by flow cytometry (AI). Data represent mean ± SEM (n = 8). Similarly, the presence of FPR1 in human isolated platelet lysates was confirmed by immunoblot analysis using selective antibodies (AII). B, the absence of *Fpr1* was confirmed in isolated platelet lysates obtained from *Fpr1* ^*−/−*^ in comparison to the control and *Fpr2/3* ^*−/−*^ mice by immunoblot analysis using selective antibodies (BI). Isolated platelets obtained from control, *Fpr1* ^*−/−,*^ and *Fpr2/3* ^*−/−*^ mice were preincubated with 10 μmol/L FITC‐conjugated fMLF and their level of binding to the platelet surface was measured by flow cytometry (BII). Data represent mean ± SEM (n = 5). C, different concentrations of fMLF were used to determine their impact on platelet activation in human isolated platelets by quantifying the level of fibrinogen binding (CI) and P‐selectin exposure (CII) using flow cytometry. Data represent mean ± SEM (n = 10). D, different concentrations of fMLF were used to determine their effects on platelet activation in human platelet‐rich plasma (PRP) by quantifying the level of fibrinogen binding (DI) and P‐selectin exposure (DII) using flow cytometry. Data represent mean ± SEM (n = 10). The blots shown are representative of three separate experiments. Protein 14‐3‐3ζ was detected as a loading control in the immunoblots. The statistical significance was calculated by 1‐way ANOVA followed by Bonferroni's correction in most of the experiments except the data shown in panel A, which were analyzed by 2‐tailed unpaired Student *t* test (*P <* 0*.01, **P <* 0*.001, and ***P <* 0*.0001). FITC, fluorescently labeled; fMLF, *N*‐formyl‐methionyl‐leucyl‐phenylalanine; FPR1, N‐formyl peptide receptor‐1

Recently, we have reported the presence of FPR2/ALX in human and its orthologue, *Fpr2/3* in human platelets.^16^ Similarly, the expression of FPR1 in human platelets at protein level, and in megakaryocytes at transcript level, has been previously reported.^15^ Furthermore, the presence of FPR1 transcripts in human and mouse platelets was demonstrated.^22^ In line with these findings, here we confirmed the presence of FPR1 on the surface of human isolated platelets by flow cytometry and in isolated platelet lysates using immunoblot analysis. Notably, the activation of platelets using 1 μg/mL cross‐linked collagen‐related peptide (CRP‐XL) increased the level of FPR1 on the surface as determined by flow cytometry (Figure 1AI), while the level of proteins identified by immunoblots remained unchanged (Figure 1AII). These data confirm the presence of FPR1 in platelets, possibly in granules or the open canalicular system or both, and their increase on the surface upon activation similar to FPR2/ALX.^16^

### 3.2 *N*‐formyl‐methionyl‐leucyl‐phenylalanine selectively binds to FPR1 on the platelet surface

A fluorescently‐labeled fMLF was used to investigate its binding to FPR1 on the surface of platelets by flow cytometry. To ascertain the selective binding of fMLF to FPR1, platelets obtained from *Fpr1* ^*−/−*^ and *Fpr2/3* ^*−/−*^ (an orthologue of human FPR2/ALX) mice along with their controls were used in this assay. The absence of *Fpr1* in platelets obtained from *Fpr1* ^*−/−*^ mice was confirmed by immunoblot analysis (Figure 1BI). The level of *Fpr1* identified in *Fpr2/3* ^*−/−*^ mouse platelets was found to be same as the controls. Notably, the absence of *Fpr2/3* protein in platelets obtained from *Fpr2/3* ^*−/−*^ mice was confirmed previously.^16^ The binding of FITC‐fMLF (5 μmol/L) was significantly reduced in *Fpr1* ^−/−^ mouse isolated platelets compared to the control and *Fpr2/3* ^*−/−*^ mouse platelets (Figure 1BII). These data confirm the selective binding of fMLF to *Fpr1* in mouse platelets.

### 3.3 *N*‐formyl‐methionyl‐leucyl‐phenylalanine stimulates platelet activation

**Figure 2:**
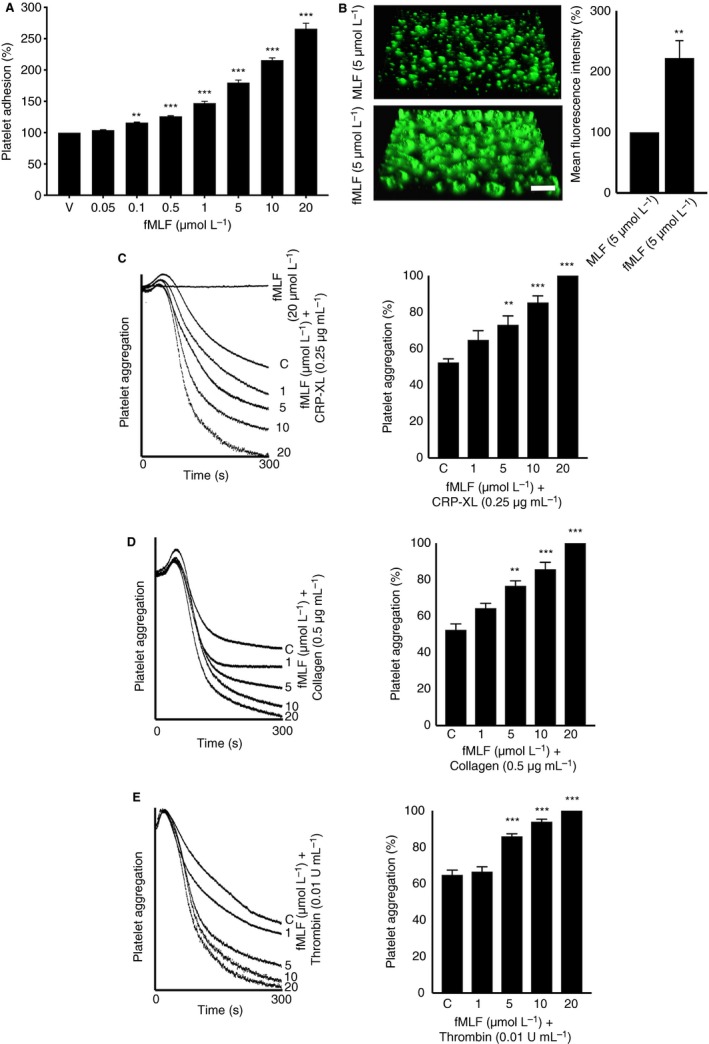
The impact of fMLF on platelet adhesion and aggregation. A, different concentrations of fMLF were used to analyze their influence on human platelet adhesion on immobilized collagen under static conditions. Data represent mean ± SEM (n = 10). B, the effect of fMLF on the modulation of thrombus formation was analyzed by using human DiOC6‐labeled whole blood that was preincubated with nonformylated MLF or fMLF (5 μmol/L) for 10 min prior to perfusion over collagen‐coated (400 μg/mL) Vena8™ Biochips. Images shown are representative of three separate experiments (10× magnification; scale bar ‐ 10 μm). Likewise, the effects of fMLF on C, cross‐linked collagen‐related peptide (CRP‐XL)‐induced, , D, collagen‐induced, or E, thrombin‐induced platelet activation were measured using isolated human platelets by optical aggregometry. Data represent mean ± SEM (n = 5). The statistical significance was calculated by 1‐way ANOVA followed by Bonferroni's correction in most of the experiments except the data shown in panels B, which were analyzed by 2‐tailed unpaired Student *t* test (*P <0.01, **P <0.001, and ***P <0.0001). fMLF, *N*‐formyl‐methionyl‐leucyl‐phenylalanine; MLF, methionyl‐leucyl‐phenylalanine

To determine whether fMLF is able to stimulate platelet activation upon binding to FPR1, a range of platelet functional assays were performed. Platelet activation triggers inside‐out signaling to integrin αIIbβ3 on the platelet surface and converts it to a high‐affinity state to allow fibrinogen binding and subsequent platelet aggregation.^23^ To examine whether fMLF influences the inside‐out signaling to integrin αIIbβ3 in platelets, the level of fibrinogen binding on the platelet surface was measured as a marker for inside‐out signaling to integrin αIIbβ3. Indeed, fMLF has increased the level of fibrinogen binding in human isolated platelets in a concentration‐dependent manner (Figure 1CI). A minimum concentration of 0.5 μmol/L fMLF has shown significant increase in fibrinogen binding compared to the resting platelets. Similarly, the level of P‐selectin exposure on the platelet surface was measured as a marker for α‐granule secretion by flow cytometry. The results indicate that fMLF has induced α‐granule secretion in human isolated platelets in a concentration‐dependent manner (Figure 1CII). Similar to the isolated platelets, fMLF has increased the level of fibrinogen binding and P‐selectin exposure in human platelets when PRP was used in a concentration‐dependent manner (Figure 1D). In addition, fMLF has increased the level of platelet adhesion to immobilized collagen under static conditions (Figure 2A). Together, these data confirm that the fMLF significantly primes platelet activation and adhesion.

### 3.4 *N*‐formyl‐methionyl‐leucyl‐phenylalanine augments thrombus formation

Microbial infection and various inflammatory diseases including sepsis are associated with the risk of disseminated intravascular coagulation or thrombosis in the microvasculature.^24^ To investigate whether fMLF has a direct impact on thrombosis, its effect on thrombus formation under arterial flow conditions was analyzed. Human DiOC_6_‐labeled whole blood was preincubated with a control (nonformylated peptide, MLF) or fMLF (5 μM) for 10 minutes prior to perfusion over collagen‐coated Vena8™ biochips. Thrombus formation was monitored for 10 minutes by acquiring fluorescent images at every 30 s . Indeed, fMLF has increased the mean fluorescence intensity of thrombi by approximately 60% compared to the controls (Figure 2B). These data demonstrate the direct impact of fMLF on augmenting the thrombus formation under arterial flow conditions in human whole blood. The effect of fMLF on other blood cells, mainly leukocytes, and their subsequent influence on thrombus formation cannot be ruled out under these circumstances.

### 3.5 Agonist‐induced platelet aggregation is amplified by fMLF

Following the determination of the effects of fMLF on thrombus formation and platelet activation, aggregation assays were performed to establish its effects on isolated platelets. Human isolated platelets were incubated with various concentrations of fMLF (1, 5, 10, and 20 μmol/L) prior to stimulation with subthreshold concentrations of different platelet agonists such as CRP‐XL (0.25 μg/mL), collagen (0.5 μg/mL), and thrombin (0.01 U/mL), and the level of aggregation was monitored for 5 minutes by optical aggregometry. Notably, fMLF has failed to induce a noticeable platelet aggregation on its own (Figure 2C) although its preincubation with platelets markedly enhanced agonist‐induced platelet aggregation. Maximum aggregation (100%) was obtained in human isolated platelets that were treated with 20 μmol/L fMLF and 0.25 μg/mL CRP‐XL for 5 minutes (Figure 2C). Similar results were obtained with collagen‐induced (Figure 2D) and thrombin‐induced (Figure 2E) platelet aggregation. These data confirm the ability of fMLF to prime platelets and amplify their aggregation upon stimulation with different agonists although it was unable to aggregate platelets on its own under the current settings.

### 3.6 *N*‐formyl‐methionyl‐leucyl‐phenylalanine selectively acts through FPR1 in platelets

**Figure 3:**
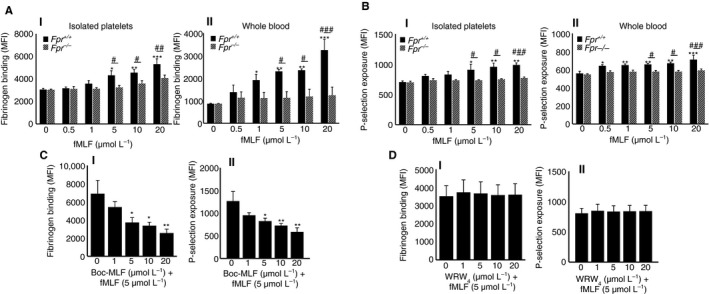
The effects of fMLF on platelet activation are largely mediated via FPR1. The platelet activation upon stimulation with various concentrations of fMLF was quantified by measuring A, the level of fibrinogen binding using FITC‐conjugated fibrinogen antibodies and P‐selectin exposure (B), using PECy5‐conjugated P‐selectin antibodies in isolated platelets (I) or whole blood (II) obtained from control and *Fpr1* ^*−/−*^ mice by flow cytometry. Data represent mean ± SEM (n = 6 for isolated platelets and n = 5 for whole blood). C, human isolated platelets were stimulated with fMLF (5 μmol/L) in the presence or absence of different concentrations of Boc‐MLF (1, 5, 10, and 20 μmol/L), and the levels of fibrinogen binding (CI) and P‐selectin exposure (CII) were analyzed by flow cytometry. Data represent mean ± SEM (n = 5). D, similarly, human isolated platelets were stimulated with fMLF (5 μmol/L) in the presence or absence of various concentrations of WRW_4_ (1, 5, 10, and 20 μmol/L), and the levels of fibrinogen binding (DI) and P‐selectin exposure (DII) were analyzed by flow cytometry. Data represent mean ± SEM (n = 5). *Represents the significant difference between the various concentrations of fMLF within the *Fpr1* ^*+/+*^ group. #Represents the significant difference between *Fpr1* ^*+/+*^ and *Fpr1* ^*−/−*^ groups. The statistical significance was calculated by 2‐way ANOVA followed by Bonferroni's correction in most of the experiments except the data shown in panel C and D, which were analyzed by 1‐way ANOVA followed by Bonferroni's correction (*P <* 0*.01. **P <0.001, and ***P <* 0*.0001). FITC, Fluorescein isothiocyanate; fMLF, *N*‐formyl‐methionyl‐leucyl‐phenylalanine; FPR1, N‐formyl peptide receptor‐1

A large number of studies indicate that fMLF binds primarily to FPR1 and exerts its effects in immune cells.^25^, ^26^, ^27^ The functional dependence of fMLF on FPR1 in platelets was determined using a pharmacological inhibitor for FPR1, Boc‐MLF in human platelets, and platelets obtained from *Fpr1* ^*−/−*^ mice through measuring the levels of fibrinogen binding and P‐selectin exposure by flow cytometry. Similar to human platelets, fMLF increased the level of fibrinogen binding [Figure 3AI (isolated platelets) and AII (whole blood)] and P‐selectin exposure [Figure 3BI (isolated platelets) and BII (whole blood)] on platelets obtained from control mice. However, the level of platelet activation by fMLF was significantly reduced in *Fpr1* ^*−/−*^ mouse platelets, which demonstrates its functional dependence on FPR1. Here, the impact of leukocytes upon binding to fMLF on platelet activation cannot be ruled out in whole blood obtained from control mice. Notably, the characterization of platelets obtained from *Fpr1* ^*−/−*^ mice failed to display any defects in the size and number of platelets or the levels of major platelet receptors such as GPVI (Figure S1i), GPIbα (Figure S1ii), αIIbβ3 (Figure S1iii), and ɑ2β1 (Figure S1iv) in comparison to the control mouse platelets. To establish the functional dependence of fMLF on FPR1 in human platelets, similar assays were performed in the presence or absence of Boc‐MLF. The preincubation of human isolated platelets with different concentrations of Boc‐MLF diminished fMLF (5 μmol/L)‐induced platelet activation as measured by the levels of fibrinogen binding (Figure 3CI) and P‐selectin exposure (Figure 3CII). To determine whether fMLF also acts through FPR2/ALX, the platelet activation was measured in the presence or absence of an FPR2/ALX‐selective inhibitor, WRW_4_. The preincubation of human isolated platelets with different concentrations of WRW_4_ did not affect the level of fMLF‐induced (5 μmol/L) platelet activation as measured by the levels of fibrinogen binding (Figure 3DI) and P‐selectin exposure (Figure 3DII). Together, these data emphasize the involvement of FPR1 in the regulation of fMLF‐mediated effects in platelets.

### 3.7 Inhibition of FPR1 reduces the agonist‐induced platelet activation

**Figure 4:**
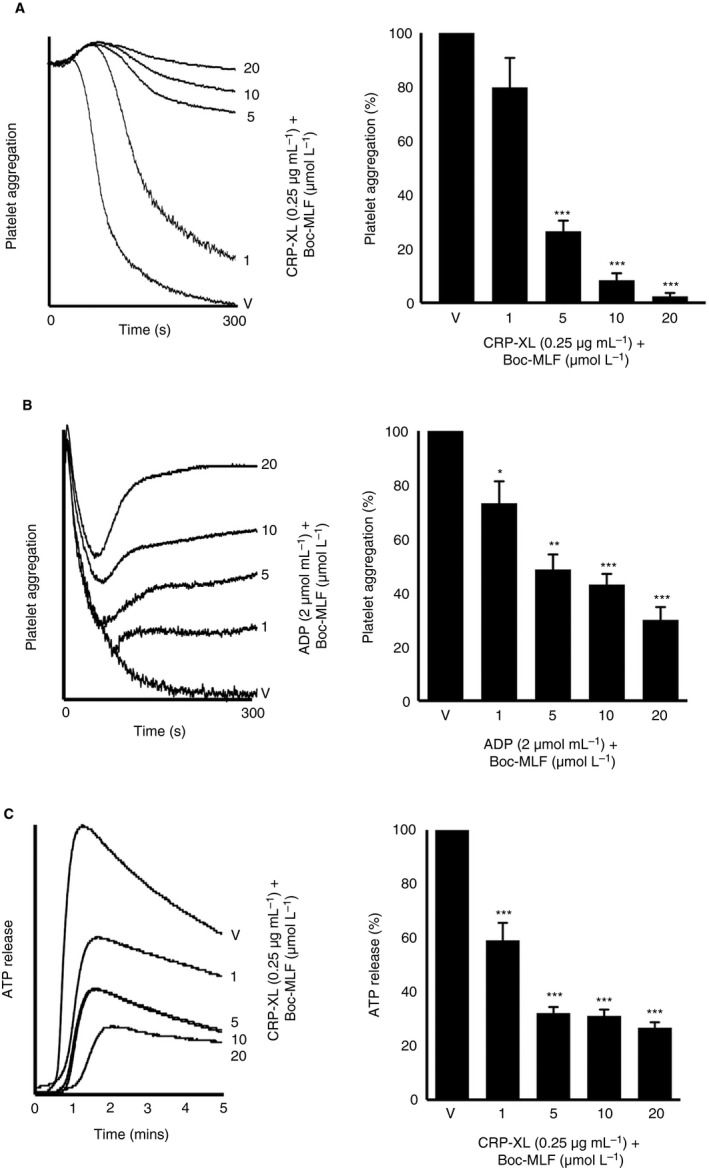
Blockade of FPR1 using a pharmacological inhibitor reduces agonist‐induced platelet aggregation. The effect of different concentrations of Boc‐MLF on CRP‐XL (0.25 μg/mL)‐induced aggregation using human isolated platelets (A) or ADP (2 μmol/L)‐induced aggregation using human platelet‐rich plasma (B) was analyzed by optical aggregometry. The level of aggregation obtained with the vehicle control was taken as 100% to calculate the extent of inhibition with Boc‐MLF‐treated samples. Data represent mean ± SEM (n = 3). Similarly, the level of ATP secretion in human isolated platelets upon activation with CRP‐XL (0.25 μg/mL) in the presence and absence of different concentrations of Boc‐MLF was measured by lumiaggregometry (C). Data represent mean ± SEM (n = 3). *P* values shown are as calculated by 1‐way ANOVA followed by Bonferroni's correction. (*P <* 0*.01, **P <* 0*.001, and ***P <0.0001). ATP, adenosine triphosphate; CRP‐XL, cross‐linked collagen‐related peptide; FPR1, N‐formyl peptide receptor‐1; MLF, methionyl‐leucyl‐phenylalanine

**Figure 5:**
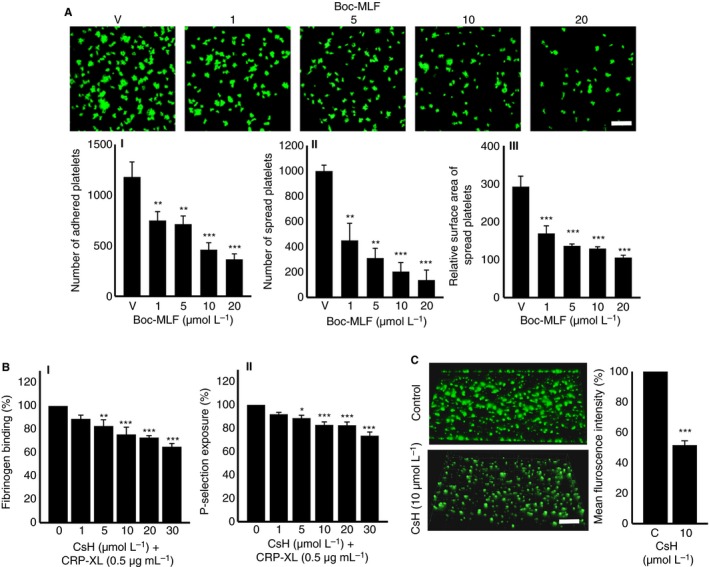
Blockade of FPR1 using a pharmacological inhibitor reduces platelet spreading, activation and thrombus formation. A, platelet adhesion and spreading on fibrinogen‐coated glass surface was analyzed in the absence or presence of Boc‐MLF (1, 5, 10, and 20 μmol/L) by confocal microscopy (60× magnification; scale bar ‐ 10 μm). The number of adhered (AI) and spread platelets (AII) and the relative surface area of spread platelets (AIII) were determined by analyzing the images using ImageJ. Ten random fields of view were recorded for each sample. Data represent mean ± SEM (n = 3). B, the levels of fibrinogen binding (BI) and P‐selectin exposure (BII) were analyzed in human platelet‐rich plasma (PRP) by flow cytometry upon stimulation with CRP‐XL (0.5 μg/mL), in the presence and absence of different concentrations of CsH (1, 5, 10, 20, and 30 μmol/L). Data represent mean ± SEM (n = 3). C, the impact of CsH on the modulation of thrombus formation was analyzed using human DiOC6‐labeled whole blood that was preincubated with a vehicle control or 10 μmol/L CsH for 10 min prior to perfusion over collagen‐coated (400 μg/mL) Vena8™ Biochips. Images shown are representative of three separate experiments (10× magnification; scale bar ‐ 10 μm). Data represent mean ± SEM (n = 3). *P* values shown are as calculated by 1‐way ANOVA followed by Bonferroni's correction except for data shown in C, which were analyzed by 2‐tailed unpaired Student *t* test, respectively. (*P <* 0*.01, **P <* 0*.001, and ***P <* 0*.0001) CRP‐XL, cross‐linked collagen‐related peptide; CsH, cyclosporin H; MLF, methionyl‐leucyl‐phenylalanine

In order to study the importance of FPR1 in the regulation of normal platelet activation, further experiments were performed using human isolated platelets in the presence or absence of Boc‐MLF. The CRP‐XL (0.25 μg/mL)‐induced platelet aggregation was significantly reduced in the presence of different concentrations of Boc‐MLF (1, 5, 10, and 20 μmol/L). For example, the addition of Boc‐MLF (20 μmol/L) reduced the platelet aggregation by around 89% (Figure 4A). Similar results were obtained with ADP‐induced platelet aggregation, wherein Boc‐MLF (20 μmol/L) reduced aggregation by approximately 82% (Figure 4B). Moreover, CRP‐XL (0.25 μg/mL)‐induced dense granule secretion as evidenced by the ATP release was significantly reduced by Boc‐MLF (Figure 4C). Similarly, to determine whether FPR1 has any influence on the outside‐in signaling triggered by integrin αIIbβ3, platelet spreading assay was performed on fibrinogen‐coated glass surfaces and analyzed using confocal microscopy. The preincubation of platelets with Boc‐MLF (1, 5, 10, and 20 μmol/L) significantly decreased the number of adhered (Figure 5AI) and spread (Figure 5AII) platelets, and the relative surface area of spreading on fibrinogen‐coated surfaces (Figure 5AIII) indicating a role for FPR1 in the modulation of integrin αIIbβ3‐mediated outside‐in signaling in platelets. In order to corroborate the involvement of FPR1 in the regulation of platelet function, CsH, an inverse agonist for FPR1, was employed. The CsH inhibited CRP‐XL (0.5 μg/mL)‐induced human isolated platelet activation as measured by the levels of fibrinogen binding (Figure 5BI) and P‐selectin exposure (Figure 5BII). Furthermore, CsH decreased the mean fluorescence intensity of thrombi in human whole blood under arterial flow conditions by approximately 60% compared to the controls (Figure 5C). These results highlight the prominent roles of FPR1 in the regulation of normal platelet function.

### 3.8 Deletion of FPR1 affects mouse platelet activation

**Figure 6:**
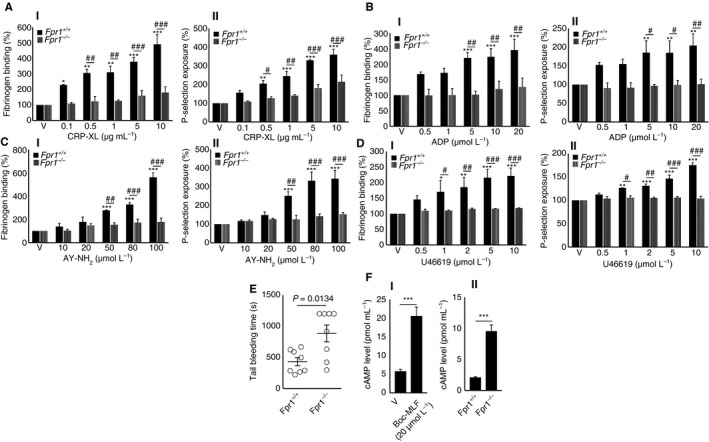
Deletion of *Fpr1* in mice reduces the agonists‐induced platelet activation and affects hemostasis. The levels of fibrinogen binding (I) and P‐selectin exposure (II) were analyzed in isolated platelets obtained from control or *Fpr1* ^*−/−*^ mice upon stimulation with various concentrations of agonists such as CRP‐XL (A), ADP (B), AY‐NH_2_ (C), or U46691 (D), by flow cytometry. Data represent mean ± SEM (n = 4). E, the impact of FPR1 on the modulation of hemostasis was analyzed by tail bleeding assay in control or *Fpr1* ^*−/−*^ mice. Data represent mean ± SEM (n = 8). F, the level of cAMP in human isolated platelets in the presence or absence of Boc‐MLF (I), and in control and *Fpr1* ^*−/−*^ mouse isolated platelets (II), was analyzed using a cAMP assay kit. *Represents the significant difference between the various concentrations of agonists within the *Fpr1* ^*+/+*^ group. ^#^Represents the significant difference between *Fpr1* ^*+/+*^ and *Fpr1* ^*−/−*^ groups. Data represent mean ± SEM (n = 4). The statistical significance was calculated by 2‐way ANOVA followed by Bonferroni's correction in most of the experiments except for the data shown in panels E and F, which were calculated by non parametric Mann‐Whitney test, and 2‐tailed unpaired Student *t* test, respectively (*P <0.01, **P <0.001, and ***P <0.0001). ADP, adenosine diphosphate; cAMP, cyclic adenosine monophosphate; CRP‐XL, cross‐linked collagen‐related peptide; FPR1, N‐formyl peptide receptor‐1

To examine further the impact of FPR1 in platelets, the whole blood obtained from control and *Fpr1* ^*−/−*^ mice was used to assess the platelet activation upon stimulation with a range of conventional platelet agonists by measuring the levels of fibrinogen binding and P‐selectin exposure using flow cytometry. Similar to the results obtained with human platelets (Figure 4), the level of (I) fibrinogen binding and (II) P‐selectin exposure in platelets obtained from *Fpr1* ^*−/−*^ mice upon stimulation with CRP‐XL (Figure 6A), ADP (Figure 6B), AY‐NH_2_ (a PAR4 agonist) (Figure 6C), and U46619, an analog of TXA_2_ (Figure 6D) was largely reduced compared to their controls. To determine the influence of FPR1 on the modulation of hemostasis, tail bleeding assay was performed in control and *Fpr1*‐deficient mice. A mean bleeding time of 429 s was observed in the control group; however, *Fpr1* ^*−/−*^ mice had a significantly increased bleeding time to a mean of 1128 s (Figure 6E). These data indicate the importance of FPR1 in the modulation of platelet function and the maintenance of hemostasis under physiological conditions. Moreover, these results are similar to the data that were reported for *Fpr2/3* ^*−/−*^ mice^16^ emphasizing the physiological significance of FPRs in the modulation of platelet function and hemostasis.

### 3.9 The inhibition or deletion of FPR1 increases the level of cyclic AMP

Cyclic AMP (cAMP) is a potent inhibitor of platelet function and its level is generally reduced upon platelet activation. Stimulants of cAMP generation are known to inhibit platelet activation.^28^ The FPRs are G_i_ protein‐coupled receptors,^29^ which are known to inhibit adenylate cyclase and thus, lead to a reduction in cAMP levels. Therefore, the deletion of genes for G_i_‐coupled receptors in mice generally increases the basal levels of cAMP in target cells.^30^, ^31^ To investigate whether the effects of FPR1 in platelets are driven through cAMP‐dependent signaling, the level of cAMP was quantified in platelets using a cAMP assay kit. The inhibition of FPR1 in human isolated platelets with Boc‐MLF (20 μmol/L) significantly elevated the level of cAMP compared to the controls (Figure 6FI). Similarly, *Fpr1* ^*−/−*^ mouse isolated platelets exhibited elevated basal levels of cAMP compared to the control mouse platelets at resting conditions (Figure 6FII). These data illustrate that the level of cAMP may play a key role in the regulation of FPR1‐mediated function in platelets.

## 4 DISCUSSION

*N*‐formyl peptides are released from bacteria or mitochondria of damaged cells.^32^, ^33^ They have been demonstrated to play substantial roles in the initiation of chemotaxis and subsequent inflammatory responses in immune cells including monocytes, mast cells, eosinophils, and neutrophils via FPRs.^9^ Despite their ability to mediate innate immune responses, they have been associated with the pathogenesis of microbial infection and inflammatory diseases. Notably, *E coli*‐derived fMLF^34^ has been implicated in bacterial cystitis,^35^ pneumococcal pneumonia,^36^ inflammatory bowel disease,^37^ pouchitis, colitis^38^ and juvenile peridontitis.^39^ It has been shown that inhalation or injection of fMLF can cause bronchial inflammation^40^ and induce rapid neutropenia, thereby increasing susceptibility to infection.^41^ The plasma levels of fMLF were increased in these conditions, and also in high‐fat‐diet‐treated mice due to altered microbiome where it impaired the glucose tolerance and insulin secretion.^42^ Many of these infectious and inflammatory conditions are associated with a risk for thrombosis and other platelet‐mediated complications.^43^, ^44^, ^45^ The concentrations of fMLF in the intestinal milieu have been reported to be at least in micromolar ranges.^46^ In this study, we demonstrate that fMLF is able to prime platelets and augment thrombus formation in micromolar concentrations. Therefore, the increased levels of fMLF under the aforementioned pathological conditions may lead to platelet activation and contribute to thrombotic complications.

The activation of platelets facilitates their adhesion to leukocytes and leads to the formation of platelet‐leukocyte aggregates (PLAs).^47^ P‐selectin on the surface of activated platelets drives the formation of PLAs via binding to P‐selectin glycoprotein ligand‐1 on the surface of leukocytes.^48^ In addition, the fibrinogen binding to integrin α_IIb_β_3_ in platelets and Mac‐1 in neutrophils was suggested to play an important role in the formation of PLAs.^49^ The PLAs are known to amplify thrombotic and proinflammatory responses in diverse inflammatory settings.^50^ Indeed, fMLF has been reported to induce PLA formation^51^ through fibrinogen binding in platelets and neutrophils.^49^ Moreover, previous studies have reported the aggregation of platelets in response to fMLF‐induced neutrophil stimulation^51^, ^52^, ^53^ through release of platelet‐activating factor^54^ and cathepsin G.^55^, ^56^ In line with these, we demonstrate that fMLF has failed to induce aggregation of isolated platelets although the pretreatment of platelets with fMLF augmented agonist‐induced aggregation. Furthermore, fMLF augmented thrombus formation under arterial flow conditions in whole blood. Given that fMLF is able to upregulate the expression of adhesion molecules^57^, ^58^ and aggregate neutrophils,^49^, ^53^ its effects on thrombus formation may be partly attributed to its interactions with leukocytes. Therefore, fMLF‐induced fibrinogen binding and P‐selectin exposure in platelets may directly trigger PLA formation as detailed previously.^59^ Although in this study the direct impact of fMLF on platelet‐mediated inflammatory responses was not analyzed, the role of fMLF cannot be excluded in such responses. Together, these data demonstrate a prominent priming role for fMLF in platelets, which may augment thrombotic and proinflammatory responses through interactions with leukocytes in pathological settings.

Formyl peptide receptor 1 is a chemoattractant receptor that is widely expressed in various cell types including neutrophils, macrophages, and platelets.^15^ Despite its well‐characterized role in the modulation of inflammatory responses, the role of FPR1 in the regulation of platelet function is poorly studied. Here, we report a crucial role for FPR1 on the regulation of platelet activation, hemostasis, and thrombosis. In line with a previous study,^15^ we confirm the presence of this receptor in human platelets by immunoblot analysis, and its upregulation upon activation of platelets with an agonist. By using selective pharmacological inhibitor, Boc‐MLF and an inverse agonist, cyclosporin H (CsH), the significance of FPR1 in the regulation of fMLF‐induced and agonist‐induced (such as CRP‐XL and ADP) platelet activation was established. The blockade of FPR1 resulted in reduced ATP release upon activation with CRP‐XL, which not only affects secondary platelet activation but may also influence the modulation of inflammatory responses.^60^ Similarly, the inhibition of FPR1 impaired the ability of platelet spreading on fibrinogen, which is essential for thrombosis and subsequent wound repair.^61^ The specificity of inhibitors to FPR1 may be limited by their ability to bind FPR2/ALX nonspecifically at higher concentrations. Therefore, the prominent role of FPR1 on the regulation of platelet activation was also corroborated using platelets obtained from *Fpr1*‐deficient mice, wherein the effects of fMLF and platelet agonists were largely diminished. The selective binding of fMLF to *Fpr1* was confirmed using platelets obtained from *Fpr1*‐deficient mice. In addition, the hemostasis in *Fpr1*‐deficient mice was affected further emphasizing its significance in the regulation of platelet function. *Fpr1*‐deficient mice displayed severe inflammation, higher mortality during pneumococcal meningitis,^62^ increased susceptibility to *Listeria monocytogenes* infection, and impaired antibacterial host defense.^17^, ^63^ In addition, *Fpr1*‐deficient mice demonstrate major roles in sterile inflammation triggered by mitochondrial *N*‐formyl peptides as demonstrated by the attenuation of inflammation in response to sterile acute lung injury in these mice.^64^ In line with these studies, here we demonstrate the dysfunction of platelets and affected hemostasis in *Fpr1*‐deficient mice, which may also substantiate the reduced inflammatory responses due to significant contribution of platelets in inflammation and innate immunity.

As a Gi protein‐coupled receptor, FPR1 triggers downstream signaling via various molecules such as phospholipase C, PI3K/AKT, and MAPK, and modulates calcium mobilization in neutrophils.^13^ The ability of fMLF to induce calcium mobilization in platelets has been previously reported.^15^ Here, we report the impact of FPR1 on cAMP‐mediated signaling in platelets using Boc‐MLF and platelets obtained from *Fpr1*‐deficient mice. Indeed, the inhibition of FPR1 in human platelets or its deletion in mouse platelets resulted in elevated levels of cAMP, which is a potent inhibitor for platelet activation. This confirmed the involvement of cAMP‐dependent signaling in the regulation of FPR1‐mediated effects in platelets.

Given the significance of FPRs and their ligands under various clinical settings, the therapeutic potential of these are being widely investigated. Notably, honokiol, a plant‐derived bioactive agent, has recently been demonstrated to reduce the proinflammatory responses induced by fMLF in neutrophils through inhibiting FPR1.^65^ Recently, we have reported the presence of FPR2/ALX in platelets, and its significance in the activation of platelets and thrombus formation upon ligation with an antimicrobial cathelicidin, LL37.^16^ In conclusion, similar to LL37, this study demonstrates a prominent role for fMLF for priming platelet activation and augmenting thrombus formation under arterial flow conditions. Using an FPR1 selective inhibitor and *Fpr1*‐deficient mice, the functional dependence of fMLF on this receptor was established. Therefore, the priming effects of fMLF on platelets may significantly contribute to the development of thrombotic and proinflammatory diatheses in pathological settings where the level of fMLF is elevated. Hence, FPR1 may act as a potential therapeutic target and its ligands may provide a powerful platform to develop novel therapeutic agents in order to treat and prevent thrombotic and inflammatory complications in diverse pathophysiological settings.

## CONFLICT OF INTEREST

The authors declare that they have no conflicts of interest with the contents of this article.

## AUTHOR CONTRIBUTIONS

Maryam F. Salamah and Sakthivel Vaiyapuri designed the study, performed experiments, analyzed data, and wrote the paper; Divyashree Ravishankar, Rajendran Vaiyapuri, and Leonardo A. Moraes performed experiments and analyzed data; Ketan Patel, Mauro Perretti, and Jonathan M. Gibbins provided guidance and support for the design of experiments and analysis of data.
