# Supplementary figures and images for: The formyl peptide fMLF primes platelet activation and augments thrombus formation
Source: J Thromb Haemost. 2019 May 24;17(7):1120–33. doi: 10.1111/jth.14466 (PMC6617722; doi:10.1111/jth.14466)

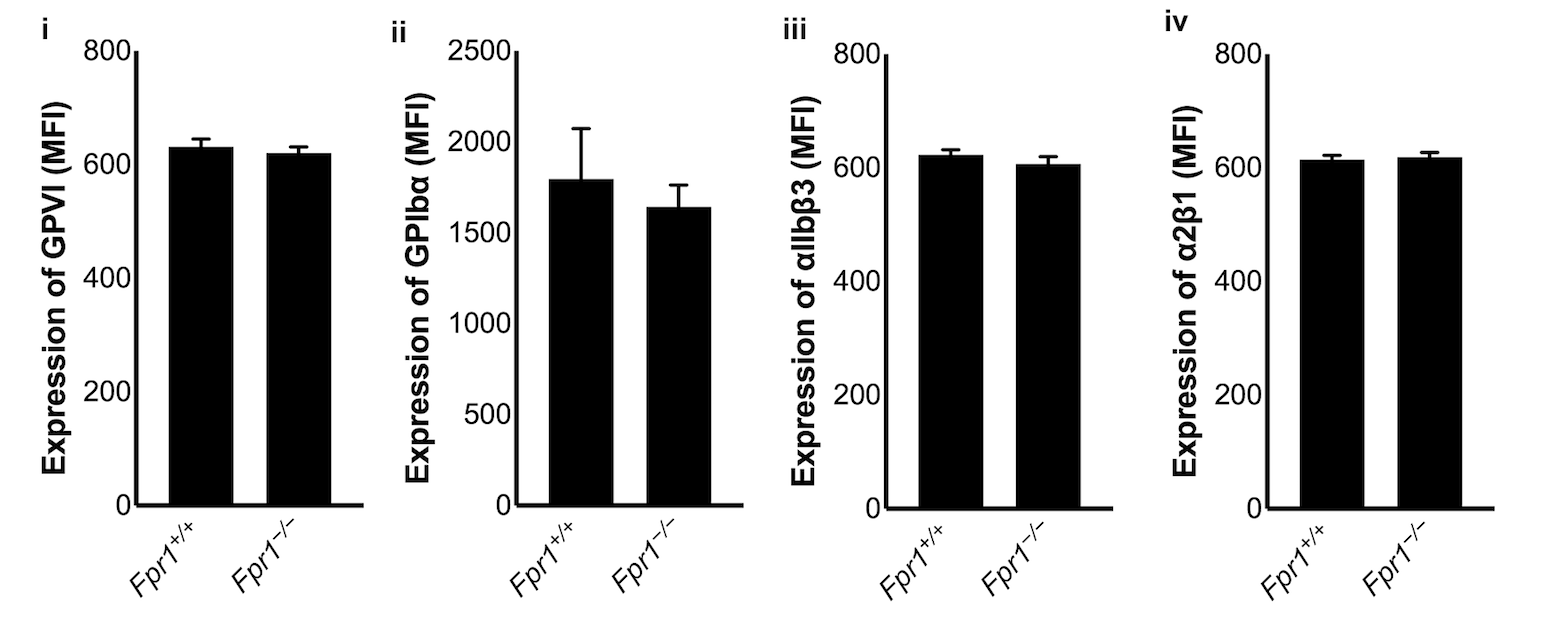

Supplement: Supplementary file 1 [file JTH-17-1120-s001.tif]
