# Supplementary material for: The formyl peptide fMLF primes platelet activation and augments thrombus formation
Source: J Thromb Haemost. 2019 May 24;17(7):1120–33. doi: 10.1111/jth.14466 (PMC6617722; doi:10.1111/jth.14466)

Supplementary Information

Methods

Flow cytometry-based assays

In order to measure the level of fibrinogen binding and P-selectin exposure on the platelet surface, flow cytometry-based assays were performed. Five microliters of PRP or isolated platelets or whole blood were incubated with 1 µl of FITC-conjugated fibrinogen antibody (1:50) (Dako, UK) and 1 µl of PECy^5-conjugated anti-CD62P (P-selectin) (1:50) antibody (BD Biosciences, UK) in the presence and absence of various concentrations of fMLF or platelet agonists. The final volume was made up to 50 µl using HEPES-buffered saline (HBS) (150mM NaCl, 5mM KCl, 1mM $MgSO_4.7H_2O$ and 10mM HEPES, pH 7.4) and the samples were incubated for 20 minutes at room temperature. Following fixation in 0.2% (v/v) formyl saline, the samples were analysed using an Accuri C6 flow cytometer (BD Biosciences, UK) by counting 5000 events within the gated population for platelets. The median fluorescence intensity was calculated using Accuri C6 software to quantify the levels of fibrinogen binding and P-selectin exposure on the surface of platelets. Similarly, for the analysis of FPR1 expression on platelets, five microliters of PRP were incubated with 1 µl of anti-FPR1 (5 µg/mL) (Novus Biological, UK) and 2 µl of Cy5-conjugated anti-mouse IgG (80 µg/mL) (Invitrogen, UK) with or without 1 µg/mL CRP-XL. Following 20 minutes incubation at room temperature, the platelets were fixed in 0.2% (v/v) formyl saline and analysed by flow cytometry. For the fMLF binding assay, following the incubation of isolated platelets with FITC-conjugated fMLF (5 µM) or vehicle control for 20 minutes, the platelets were fixed in 0.2% (v/v) formyl saline and analysed by flow cytometry.

SDS-PAGE and immunoblotting analysis

Immunoblot analysis was performed using platelet lysates that prepared under reducing conditions. The samples were heated to 90°C for 10 minutes and subjected to SDS-PAGE using 10% resolving gels. The gels were then transferred to polyvinylidene difluoride (PVDF) membranes and blocked by incubation in 5% (w/v) bovine serum albumin (BSA) in TBS-T (20mM Tris, 140mM NaCl and Tween-20, pH 7.6). Following an overnight incubation with primary anti-FPR1 antibody (1:500) (Abcam, UK), the blots were washed with TBS-T and incubated with secondary Cy5-conjugated goat anti-rabbit IgG antibodies (1:1000) (Invitrogen, UK) in TBS-T containing 5% (w/v) BSA for one hour at room temperature. Following washing in TBS-T for one hour at room temperature, the blots were analysed using a Typhoon 9400 Variable Mode Imager system (GE Healthcare, UK). Equal loading of proteins in each lane was determined using anti-human 14-3-3ζ antibodies (1:1000) (Santa Cruz Biotechnology, USA).

Cyclic nucleotide assay

A cAMP ELISA kit (Cambridge Bioscience, UK) was used for the detection of the total levels of cAMP in human and mouse platelets. Human isolated platelets were pre-incubated for 10 minutes with a selective inhibitor for FPR1, Boc-MLF. Similarly, platelets obtained from control or *Fpr1*$^{-/-}$ mice were also used. The platelets were incubated with 0.1M HCl and the levels of cAMP were calculated according to the manufacturer's protocol.

Platelet spreading assay

Isolated human platelets were treated with different concentrations of an FPR1-selective inhibitor, Boc-MLF, prior to loading onto fibrinogen (100 µg/mL)-coated coverslips and incubation for 30 minutes. The coverslips were then washed with PBS to remove non-adhered platelets. Adhered platelets were fixed with 0.2% (v/v) formyl saline for 10 minutes prior to permeabilisation with 0.2% (v/v) Triton X-100 for five minutes at room temperature. Adhered platelets were stained with Alexa Fluor 488-conjugated phalloidin for 30 minutes at room temperature. The coverslips were then mounted onto slides and analysed using a Nikon A1-R confocal microscope (60x objective). Ten random fields of view were recorded for each sample. The data were analysed to quantify the number of adhered and spread platelets, and the relative area of spread platelets using ImageJ. The relative surface area of spread platelets was obtained by subtracting the surface area of resting platelets.

Supplementary Figure 1

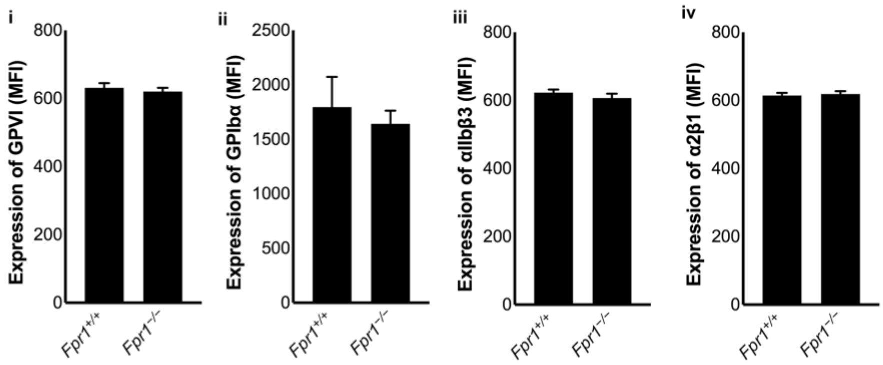

Supplement: Supplementary file 2 [file JTH-17-1120-s002.pdf]
